# Is a polymer semiconductor having a “perfect” regular structure desirable for organic thin film transistors?†
†Electronic supplementary information (ESI) available. See DOI: 10.1039/c5sc00843c



**DOI:** 10.1039/c5sc00843c

**Published:** 2015-03-31

**Authors:** Wei Hong, Shaoyun Chen, Bin Sun, Mark A. Arnould, Yuezhong Meng, Yuning Li

**Affiliations:** a Department of Chemical Engineering and Waterloo Institute for Nanotechnology (WIN) , University of Waterloo , 200 University Avenue West , Waterloo , Ontario N2L 3G1 , Canada . Email: yuning.li@uwaterloo.ca; b The Key Laboratory of Low-carbon Chemistry & Energy Conservation of Guangdong Province/State Key Laboratory of Optoelectronic Materials and Technologies , Sun Yat-Sen University , Guangzhou , 510275 , P. R. China; c Xerox Corporation , 800 Phillips Road, Bldg. 0139-64A , Webster , NY 14580 , USA; d College of Chemistry and Life Science , Quanzhou Normal University , Quanzhou , Fujian 362000 , P. R. China

## Abstract

This study utilized high temperature NMR and matrix-assisted laser desorption/ionization time-of-flight (MALDI-ToF) mass spectrometry to reveal that appreciable amounts of structural defects are present in the diketopyrrolopyrrole (DPP)–quaterthiophene copolymers (PDQT) synthesized by the Stille coupling polymerization with Pd(PPh_3_)_2_Cl_2_, Pd_2_(dba)_3_/P(*o*-tol)_3_, and Pd(PPh_3_)_4_ catalyst systems. It was proposed that these structural defects were produced *via* homocoupling side reactions of the C–Br bonds and the organostannane species. Model Stille coupling reactions further substantiated that the amount of structural defects are catalyst-dependent following the order of Pd(PPh_3_)_2_Cl_2_ > Pd_2_(dba)_3_/P(*o*-tol)_3_ > Pd(PPh_3_)_4_. To verify the structural assignments, “perfect” structurally regular PDQT polymers were prepared using Yamamoto coupling polymerization. When compared to the structurally regular polymers, the polymers containing defects exhibited notable redshifts in their absorption spectra. Surprisingly, the “perfect” structurally regular polymers showed poor molecular ordering in thin films and very low charge transport performance as channel semiconductors in organic thin film transistors (OTFTs). On the contrary, all the “defected” polymers exhibited much improved molecular ordering and significantly higher charge carrier mobility.

## Abstract 2

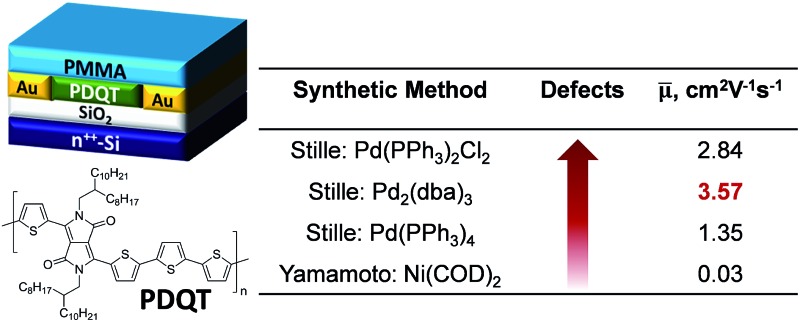
Appreciable amounts of structural defects produced during Stille coupling polymerization have unexpected beneficial effects on the molecular ordering and charge transport performance of polymers.

## Introduction

π-Conjugated polymers are considered to be promising semiconductors for organic thin film transistors (OTFTs), which have potential applications in flexible displays, radio frequency identification (RFID) tags, sensors, *etc.*^1–4^ Polymer semiconductors capable of forming highly ordered molecular organization are preferred and have been extensively pursued to achieve efficient charge transport performance,^5–9^ even though in some cases amorphous/disordered polymers could provide adequate performance.^10–16^ Conjugated polymers with well-defined repeat units have been considered desirable for OTFTs because they can self-assemble into a long-range chain packing order that would promote charge carrier transport. The best known example of this behavior is poly(3-hexylthiophene) (P3HT). The regio-irregular P3HT forms amorphous films that exhibit low charge carrier mobility (∼10^–5^ cm^2^ V^–1^ s^–1^),^17^ but the highly regio-regular, head-to-tail P3HT is crystalline and has demonstrated significantly improved mobility (up to ∼0.1 cm^2^ V^–1^ s^–1^)^5,18^ in OTFTs. Very few random copolymers have been reported for OTFTs^19,20^ since the molecular ordering of random copolymers would be hindered, resulting in lower mobility compared to the regular alternating copolymers.^19^

In recent years, polymer semiconductors having alternating electron donor (D) and acceptor (A) units on their backbone have received escalating attention due to their remarkable performance as channel semiconductors in OTFTs.^21–26^ Charge carrier mobility as high as ∼10 cm^2^ V^–1^ s^–1^ has recently been achieved.^27–30^ The excellent charge transport performance observed for these D–A polymers is believed to be brought about by the strong intermolecular D–A interaction that could shorten the co-facial π–π stacking distance between conjugated polymer main chains allowing for more efficient charge hopping.^23^ One of the most commonly used synthetic approaches to D–A polymers is the Stille coupling reaction,^31,32^ where an organoditin (or organodistannane) and an organodielectrophile (often a dihalo compound) are used as comonomers in the presence of a Pd catalyst to form a long chain polymer.

**Scheme 1:**
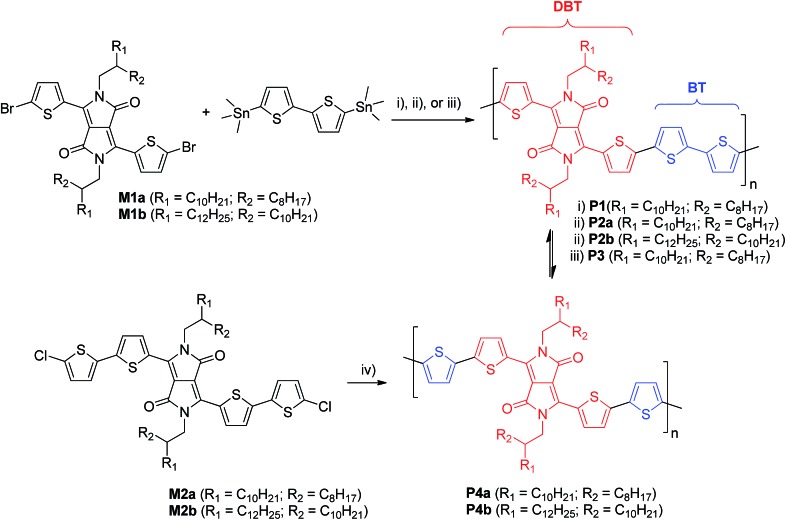
Synthetic routes to PDQT polymers **P1–P4**: (i) Pd(PPh_3_)_2_Cl_2_/toluene/reflux; (ii) Pd_2_(dba)_3_/P(*o*-tol)_3_ (molar ratio: 1/4)/chlorobenzene/130 °C; (iii) Pd(PPh_3_)_4_/toluene/reflux; (iv) Ni(COD)_2_/2,2′-bipyridyl/THF/60 °C.

Previously we reported a D–A polymer, PDQT (Scheme 1)^33–35^ having a diketopyrrolopyrrole (DPP) and a quaterthiophene in the repeat unit, which showed high hole mobility up to 6.9 cm^2^ V^–1^ s^–1^ in OTFTs. Initially this polymer (**P1**) was synthesized using the Stille coupling reaction of 3,6-bis(5-bromothiophen-2-yl)-2,5-bis(2-octyldodecyl)pyrrolo[3,4-*c*]pyrrole-1,4(2*H*,5*H*)-dione (**M1a**) and 5,5′-bis(trimethylstannyl)-2,2′-bithiophene with Pd(PPh_3_)Cl_2_ as a catalyst.^33^ In subsequent studies, we also used another catalyst system consisting of Pd_2_(dba)_3_/P(*o*-tol)_3_ (**P2**) in order to increase the molecular weight of the polymer to improve mobility.^27^ Interestingly, we noticed that the PDQT polymers synthesized with these two different catalyst systems showed dramatically different UV-Vis absorption spectra in both solution and the solid state. This suggests that they may have different backbone structures and that at least one of these two catalysts resulted in structural defects, prompting us to investigate their structural differences in more detail. The Suzuki coupling reaction^36^ is another frequently used cross-coupling polymerization technique for synthesizing conjugated polymers. A recent paper by Janssen and coworkers^37^ reported that homocoupling side reactions of the bromo compounds were observed in the Suzuki coupling reaction with a catalyst system Pd_2_(dba)_3_/PPh_3_ (with a molar ratio of 1 : 1). The presence of these structural defects had a detrimental effect on the photovoltaic performance of the polymers and should be avoided. The authors also suggested that the Stille coupling reaction with the same catalyst system might involve similar side reactions.

In this study, we investigated the structures and properties of PDQT polymers synthesized by the Stille coupling polymerization using three common catalyst systems: Pd(PPh_3_)Cl_2_, Pd_2_(dba)_3_/P(*o*-tol)_3_, and Pd(PPh_3_)_4_. Our results unambiguously demonstrate that significant amounts of structural defects were produced during the Stille coupling polymerization. It was found that these structural defects have significant impacts on the UV-Vis absorption, molecular ordering, as well as the OTFT performance of these polymers.

## Results and discussion

PDQT polymers **P1**, **P2a**, and **P3** were synthesized by the Stille coupling polymerization of the dibromo monomer **M1a** with equivalent 5,5′-bis(trimethylstannyl)-2,2′-bithiophene using three different Pd catalyst systems, Pd(PPh_3_)_2_Cl_2_, Pd_2_(dba)_3_/P(*o*-tol)_3_ (molar ratio: 1/4), and Pd(PPh_3_)_4_, as outlined in Scheme 1. Polymer **P4a** was prepared by the Yamamoto coupling polymerization^38^ of the dichloro monomer **M2a** using Ni(COD)_2_. Each repeat unit in **P4a** has one DBT and two thiophenes (T–DBT–T), which is equivalent to the repeat unit (DBT–BT) of **P1**, **P2a**, and **P3** (Scheme 1). However, **P4a** can be considered a homopolymer and only one type of repeat unit would exist in the resulting polymer. A branched long chain hydrocarbon, 2-octyldodecyl (C20), was used on the DPP unit to improve the solubility of the polymers. **P1**, **P2a**, and **P3** made *via* the Stille coupling showed good solubility in chloroform, while **P4a** made by the Yamamoto coupling is insoluble in the same solvent. A soluble fraction of **P4a** (34%) was obtained by Soxhlet extraction with 1,1,2,2-tetrachloroethane (TCE), which has a much higher boiling point (146.5 °C) than chloroform (61.2 °C). The extremely poor solubility of **P4a** is thought to be due to the highly regular structure of the polymer yielding an extremely rigid backbone. To improve the solubility, a longer side chain, 2-decyltetradecyl (C24), was used and the resulting polymer **P4b** exhibited much better solubility (62.1% and 24.8% yields were obtained from the fractions extracted with chloroform and TCE, respectively; only the fraction extracted with chloroform was used for characterization of this polymer in this study). In order to make a direct comparison with **P4b**, **P2b** with the same C24 side chain was prepared using the Pd_2_(dba)_3_/P(*o*-tol)_3_ catalyst.

The molecular weight of these polymers was measured using a high temperature gel-permeation chromatography (HT-GPC) system at 140 °C with 1,2,4-trichlorobenzene as an eluent and polystyrene as standards. The results are summarized in Table 1. All the polymers have the number average molecular weight (*M*_n_) greater than 20 kg mol^–1^. Due to the poor solubility of **P4a**, its molecular weight could not be measured with this method.

**Table 1:** Summary of molecular weights and properties of polymers **P1–P4**^*a*^

Polymer	Catalyst	HT-GPC		MALDI-ToF	*λ*_max_ (nm)		*E*_g_^opt^ (eV)	*E*_HOMO_/*E*_LUMO_ (eV)
		*M*_n_ (kg mol^–1^)	PDI	DBT : BT ratio^*b*^	Sol.	Film		
**P1**	Pd(PPh_3_)_2_Cl_2_	21.4	2.03	1 : 0.67–1.33 (*n* = 9)^*c*^	775	783	1.24	–5.3/–4.1
**P2a**	Pd_2_(dba)_3_/P(*o*-tol)_3_	47.0	2.90	1 : 0.43–1.14 (*n* = 7)	787	786	1.34	–5.3/–4.0
**P2b**	Pd_2_(dba)_3_/P(*o*-tol)_3_	54.9	3.12	NA	781	781	1.32	–5.3/–4.0
**P3**	Pd(PPh_3_)_4_	38.3	2.16	1 : 0.80–1.3 (*n* = 10)	798	787	1.38	–5.3/–4.0
**P4a** ^*d*^	Ni(COD)_2_/2,2′-bipyridyl	—	—	1 : 1	773	783	1.46	–5.3/–3.9
**P4b** ^*e*^	Ni(COD)_2_/2,2′-bipyridyl	23.8	2.51	1 : 1	785	790	1.43	–5.3/–3.9

^*a*^HT-GPC, UV-Vis, and cyclic voltammetry data for **P2a** and **P2b** were reported previously.^34,35^

^*b*^DBT: 3,6-di([2,2′-bithiophen]-5-yl)-2,5-bis(2-octyldodecyl)pyrrolo[3,4-*c*]pyrrole-1,4(2*H*,5*H*)-dione or 3,6-di([2,2′-bithiophen]-5-yl)-2,5-bis(2-tetradecyldecyl)pyrrolo[3,4-*c*]pyrrole-1,4(2*H*,5*H*)-dione, BT: 2,2′-bithiophen-diyl.

^*c*^ *n*: the number of DBT units.

^*d*^The fraction extracted with TCE.

^*e*^The fraction extracted with chloroform.

**Fig. 1:**
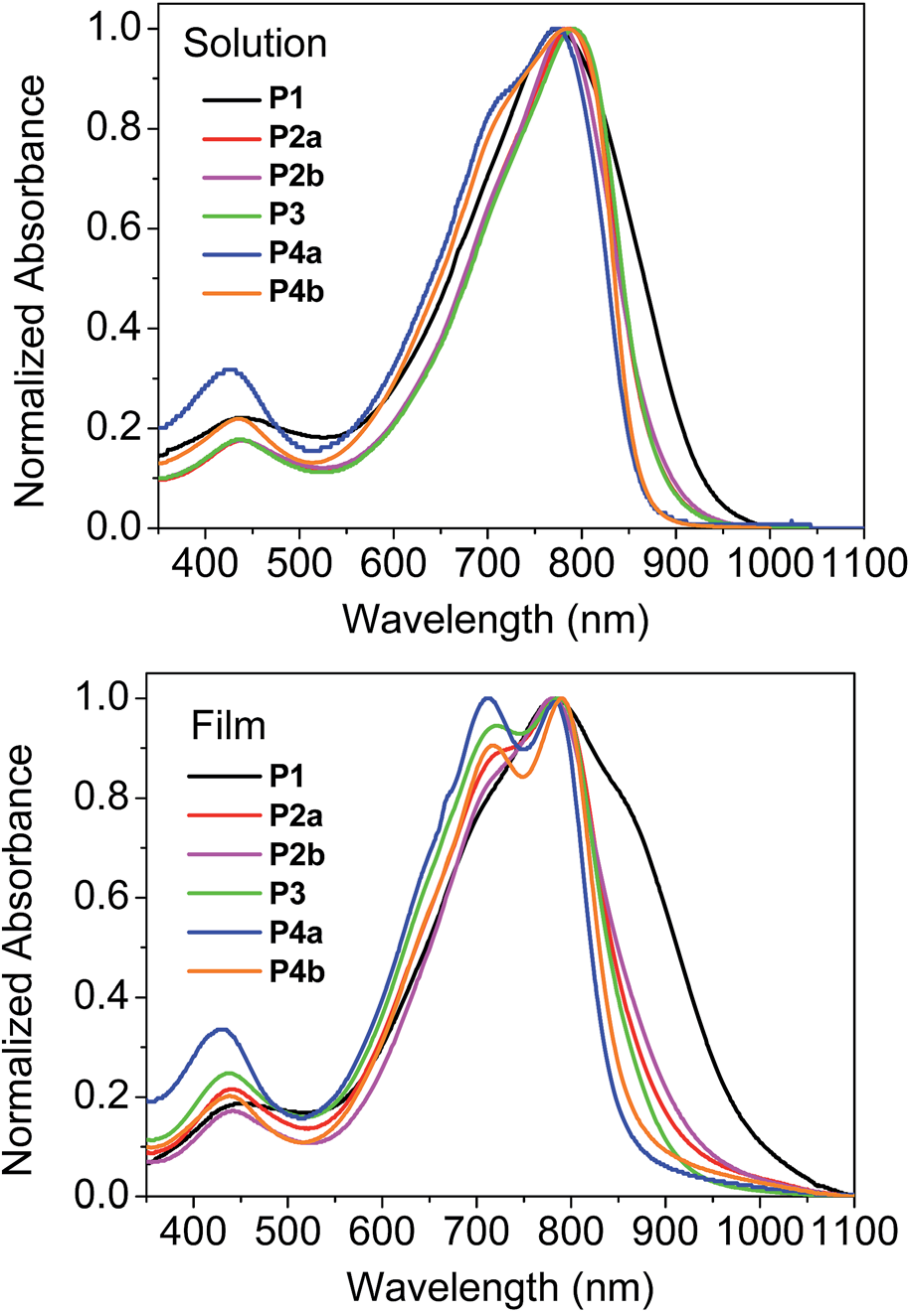
UV-Vis absorption spectra of **P1–P4** in solution (TCE) and in thin films.

The UV-Vis spectra of the polymers are shown in Fig. 1. In solution, the polymers showed the wavelengths of maximum absorption (*λ*_max_) ranging from 773 nm to 798 nm. The relatively small *λ*_max_ (773 nm) observed for **P4a** is thought to be due to the lower molecular weight of its soluble fraction in TCE. Most of the polymers showed a slight redshift (<10 nm) in *λ*_max_ for the thin film when compared with the solution spectra due to intermolecular interaction in the solid state. It was also observed that these polymers showed dramatic differences in the long wavelength absorption side of the spectra (particularly in thin films) due to the presence of homocoupled defects in the polymer backbone.^37^ (This phenomenon will be further discussed later.) **P1** displayed the most pronounced extension into the long wavelength region, followed by **P2** then **P3**, with **P4** showing the least. Therefore the optical band gaps for these polymers follow a trend of **P4** > **P3** > **P2** > **P1**. The molecular weight of the polymers, which correlates to the main chain conjugation length, would influence the band gap of the polymers if the maximum effective conjugation length is not reached. However, the differences in the molecular weight do not seem to correlate with the different absorption profiles observed for these polymers synthesized using different catalyst systems. For instance, it can be seen that **P1**, with the lowest molecular weight among these polymers (except for **P4a**), showed the largest redshift. Therefore, the main reason for the redshifts observed for **P1–P3** as compared to **P4** might be due to their potentially different backbone structures. The highest occupied molecular orbital (HOMO) energy levels of these polymers are very similar (∼–5.3 eV) as determined by cyclic voltammetry with ferrocene as a reference (Table 1). The lowest unoccupied molecular orbital (LUMO) energy levels were estimated using the *E*_HOMO_ and the optical band gaps to be around –4 eV with the highest value of –3.9 eV for **P4a** and **P4b** and the lowest value of –4.1 eV for **P1**.

**Fig. 2:**
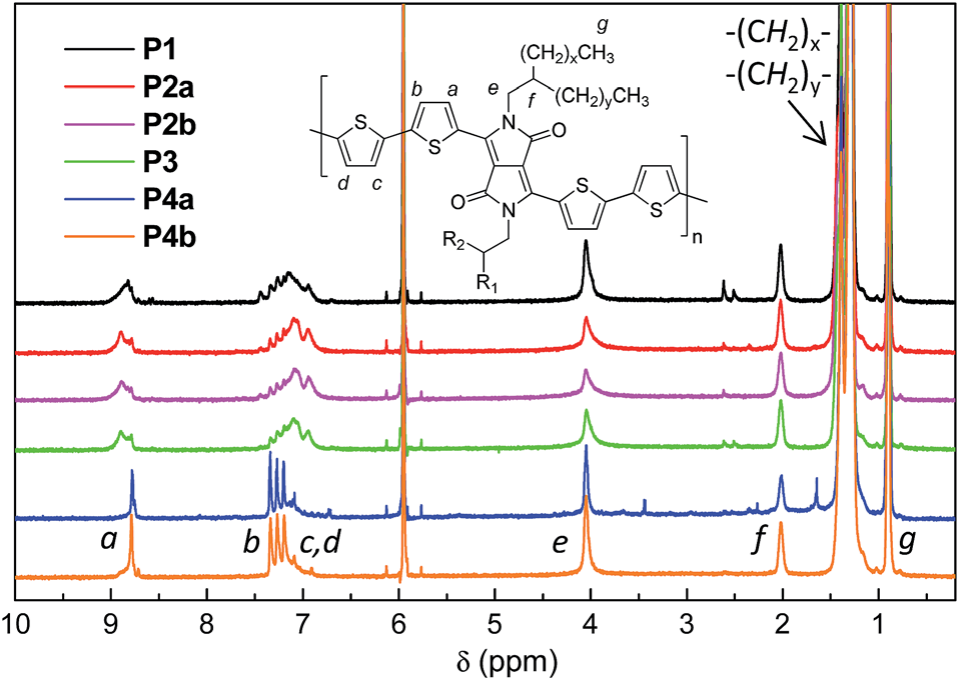
500 MHz ^1^H NMR spectra of **P1–P4** acquired in deuterated 1,1,2,2-tetrachloroethane (TCE-d_2_) at 125 °C.

^1^H NMR was utilized to investigate the molecular structures of these polymers. However ^1^H NMR spectra of all the polymers measured at room temperature in CDCl_3_ or deuterated 1,1,2,2-tetrachloroethane (TCE-d_2_) showed very broad peaks (ESI†), indicating that the polymer chains aggregated in solution, a phenomenon that has been observed for some other D–A polymers.^33,39^ The resolution of the ^1^H NMR spectra was improved by measuring the polymers in TCE-d_2_ at an elevated temperature of 125 °C to dissociate the polymer chain aggregates. This resulted in well-resolved peaks for the spectra of **P4a** and **P4b** (Fig. 2). As expected, these two polymers showed quite simple resonance peak patterns due to the presence of the exact repeat units enabled by the Yamamoto coupling polymerization. Some minor peaks are also observed and can be assigned to the end groups. Distinct peaks in the aromatic region for **P1–P3**, which correspond to those in **P4**, are observed, but several intense peaks at other positions are also present. The observation of more complex NMR patterns for **P1–P3** compared to **P4** is most likely due to their different backbone structures. This strongly suggests that side reactions occurred to form structural defects during the Stille coupling polymerization with the catalyst systems utilized in this study.

**Fig. 3:**
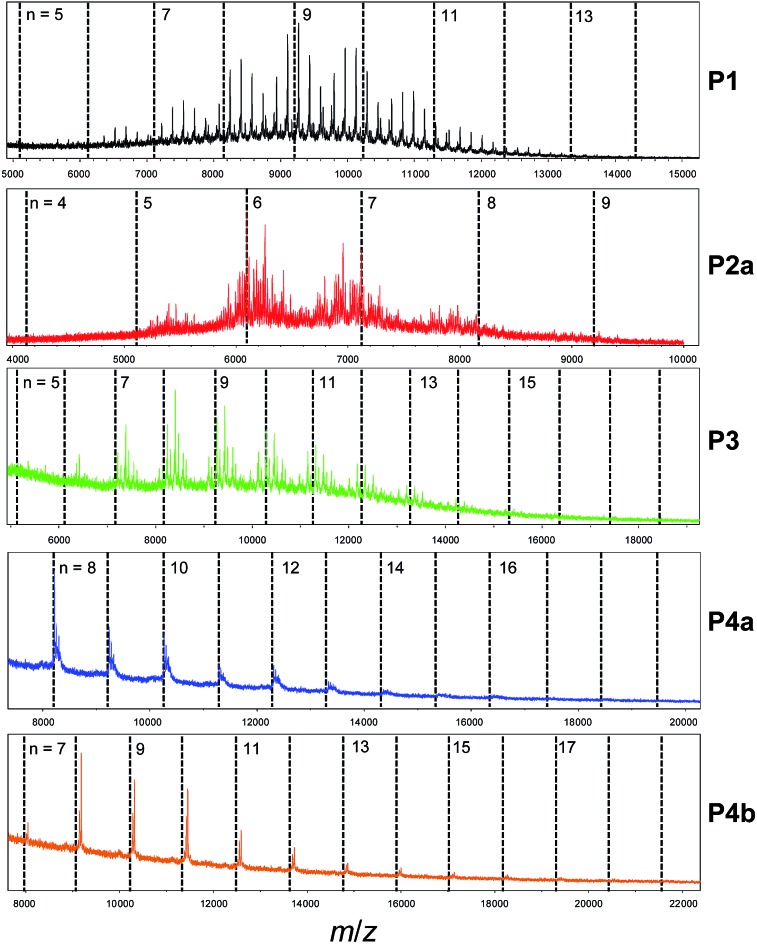
MALDI-ToF mass spectra of polymers from selected times in the chromatograms, where the vertical dashed lines represent the theoretical molecular masses of polymers with the regular structure –(DBT–BT)_*n*_– at different numbers of repeat units (*n*) without contribution of the terminal groups.

To shed more light on the polymer structures, MALDI-ToF mass spectroscopy was utilized to obtain the molecular masses for individual polymer chains after GPC separation. Fig. 3 shows that for **P4a** and **P4b** there is an excellent agreement of the measured molecular masses of the polymers with the calculated/theoretical values (represented by the vertical dashed lines), indicating their perfect polymer structures with exact repeat units. The offset from the theoretical values arises from the masses of the end groups. On the other hand, the molecular masses of **P1** are mostly not observed at the theoretical molecular masses, but appear to be distributed more like a random copolymer yielding a distribution of distributions, *i.e.*, there exist many chains containing less or more BT units than expected. There are dominant end group structures, so the spectrum is not further convoluted by overlapping end group distributions. In the spectrum of **P2a**, the distributions are more severely affected due to the extremely complex distribution of end group structures (with similar intensities) superimposed onto the broad comonomer distribution. The mass spectrum of **P3** shows a better overall match to the theoretical values as compared to **P1** and **P2a**. The broad comonomer distribution for each of the **P1**, **P2a** and **P3** samples as compared to **P4a** and **P4b** indicates that there are significant amounts of structural defects in these polymers. For example, the ratio of the two comonomer units (DBT and BT) for **P1** were calculated for polymer chains containing 9 DBT units (*n* = 9). It was found that these peaks correspond to molecules having DBT : BT ratios of 9 : 6–12 (1 : 0.67–1.33) (Table 1). The existence of polymer chains with DBT : BT ratios greater than the expected largest possible ratio of 1 : 0.89 for polymers with alternating DBT and BT units (DBT : BT = 9 : 8, 9 : 9, and 9 : 10 (or 1 : 0.89, 1 : 1, and 1.11)) suggests that some DBT units are adjoined or homocoupled. For **P2a**, polymer chains around *n* = 7 have DBT : BT ratios between 1 : 0.43 and 1 : 1.14, indicating that there exist chains having larger than the DBT : BT ratios expected for molecules with alternating DBT : BT units (7 : 6, 7 : 7, and 7 : 8 (or 1 : 0.86, 1 : 1, and 1 : 1.14)). Again, this indicates that this polymer has many DBT–DBT homocoupling units. The DBT : BT ratios of **P3** molecules around *n* = 10 are in the range of 10 : 8–13 (1 : 0.8–1.3), which are close to the expected ratios of 10 : 9, 10 : 10, and 10 : 11 (1 : 0.9, 1 : 1, and 1 : 1.1), suggesting that this polymer has less homocoupling DBT–DBT units. Polymers **4a** and **4b** showed an exact DBT : BT ratio of 1 : 1 for all the peaks since their monomers have an intrinsic DBT : BT ratio of 1 : 1.

**Scheme 2:**
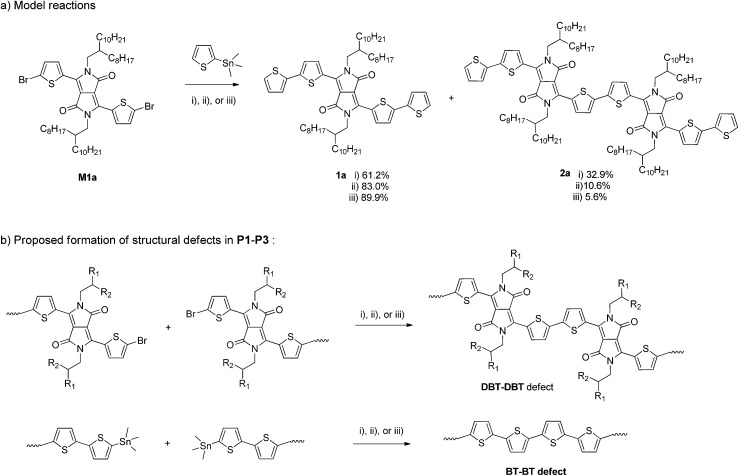
(a) Model Stille coupling reactions using different catalyst systems: (i) Pd(PPh_3_)_2_Cl_2_/toluene/reflux; (ii) Pd_2_(dba)_3_/P(*o*-tol)_3_ (molar ratio: 1/4)/chlorobenzene/130 °C; (iii) Pd(PPh_3_)_4_/toluene/reflux. (b) Proposed formation of structural defects DBT–DBT and BT–BT *via* the homocoupling side reactions.

To further investigate the possible side reactions during the Stille coupling, model reactions between **M1a** and a monostannyl compound, 2-trimethylstannylthiophene, were conducted using the three catalyst systems under the same reaction conditions for synthesizing **P1**, **P2a**, and **P3**, respectively (Scheme 2a). The expected product **1a** was isolated from the final reaction mixtures by column chromatography on silica gel with yields of 62.1%, 83.0%, and 89.9% with respect to the individual reaction conditions (i), (ii), and (iii) used for **P1**, **P2a**, and **P3**, respectively. A by-product **2a** was isolated for all three model reactions. A surprisingly large amount of **2a** (32.9% isolated yield) was obtained when Pd(PPh_3_)Cl_2_ was used as the catalyst, while 10.6% (ii) and 5.6% (iii) of compound **2a** were isolated for reactions using Pd_2_(dba)_3_/P(*o*-tol)_3_ and Pd(PPh_3_)_4_, respectively. Compound **2a** was most likely formed from the homocoupling of two C–Br bonds of two **M1a** molecules and/or the intermediate with one side capped with a thiophene, T–DBT–Br. Based on the yields of the homocoupling product **2a** for the three catalyst systems, it is reasonable to assume that the same type of side reactions involving C–Br containing species occurred during the Stille coupling polymerization (Scheme 2b). The content of the DBT–DBT homocoupling defects in the polymers would follow the order of **P1** > **P2** > **P3**, which is in agreement with the MALDI-ToF results discussed previously. Similar side reactions were reported very recently for the Suzuki coupling reaction and was also suggested to occur during the Stille coupling polymerization in a different catalyst system Pd_2_(dba)_3_/PPh_3_ (1 : 1).^37^ A homopolymer of **M1a** (PDBT-20), which contains only DBT units, was observed to have a much longer *λ*_max_ (917 nm in solution and 928 nm in thin films)^40^ than that of **P4**, suggesting that the higher the amount of the DBT–DBT homocoupling defects, the longer the absorption wavelength of the PDQT polymer. The absorption redshift trend of **P1** > **P2** > **P3** with respect to **P4** agrees well with the DBT–DBT defect contents of these polymers. The presence of the largest amount of DBT–DBT defects in **P1** also leads to its deepest LUMO energy level among the polymers studied because of the strong electron-accepting effect of the DPP units. It should be noted that other side reactions such as the homocoupling of organostannanes, which is a common side reaction observed for the Stille coupling,^41^ might also occur during the polymerization reactions in this study. This type of side reaction would form the BT–BT homocoupling defects in **P1–P3** as illustrated in Scheme 2b. This could explain the presence of polymer chains that have DBT : BT ratios smaller than expected (with more BT units than expected) for **P1** and **P3** revealed by their MALDI-ToF spectra (Table 1 and Fig. 3). **P2a** showed the smallest DBT : BT ratio 1 : 1.14 (7 : 8) at *n* = 7, indicating that less BT : BT defects present in this polymer, although the existence of the BT : BT defects in this polymer cannot be completely ruled out solely based on its DBT : BT ratios. Attempts to isolate the possible bithiophene by-product from the model Stille coupling reaction mixtures with column chromatography or detect this by-product by mass spectroscopy were unsuccessful due to the extremely low anticipated mass yields of this by-product and the interference of other impurities. Nonetheless, the almost quantitative isolated yields of **P1–P3** (97–99%) support that most of the BT units in the 5,5′-bis(trimethylstannyl)-2,2′-bithiophene monomer were incorporated into these polymers, which suggests that similarly significant amounts of homocouling BT–BT defects with respect to that of the DBT–DBT defects might be formed.

**Fig. 4:**
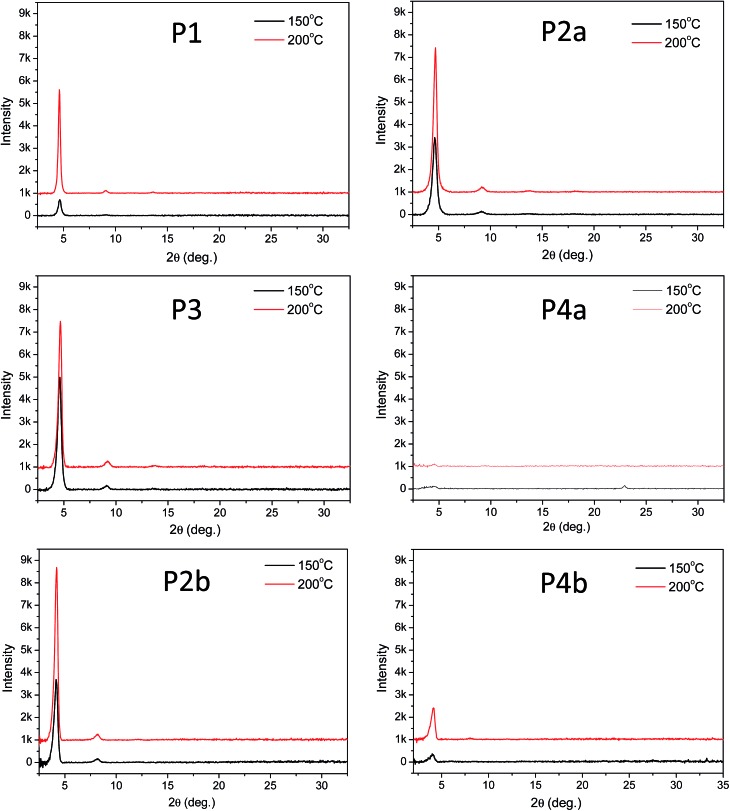
Reflective XRD diagrams of **P1–P4** thin films (with similar thicknesses of ∼50 nm) spin coated on dodecyltrichlorosilane-modified SiO_2_/Si wafer and annealed at 150 °C or 200 °C using Cu Kα1 radiation (*λ* = 0.15406 nm). Note that the intensity scale for **P4a** is one-tenth of that in other diagrams due to the low diffraction intensities of this polymer. Data for **P2a** and **P2b** were reported previously.^35^

**Fig. 5:**
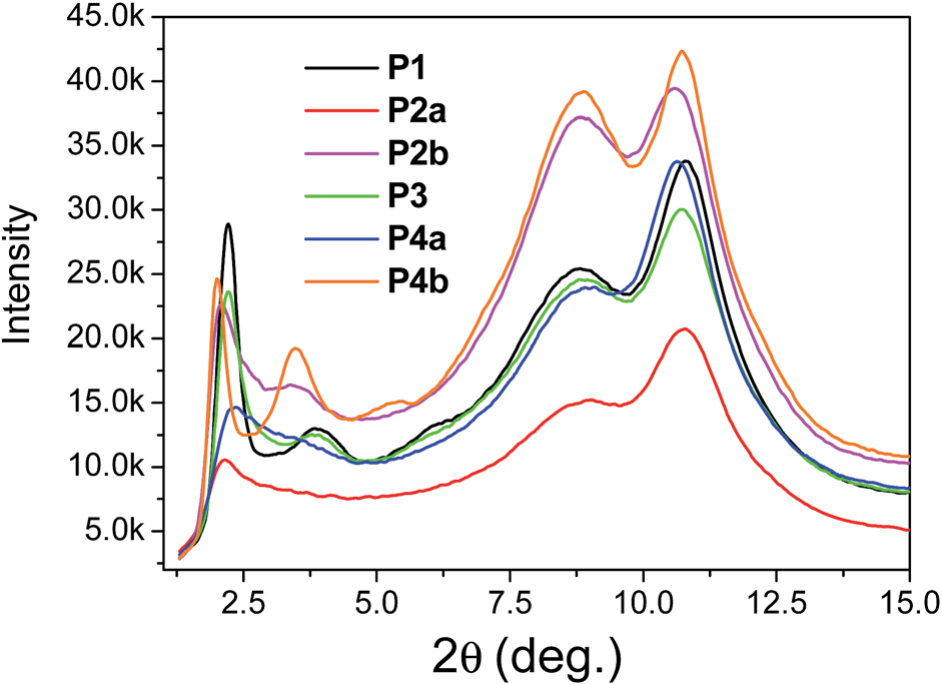
Transmission XRD diagrams of **P1–P4** flakes (thicknesses were not intentionally controlled) annealed at 200 °C using Mo Kα radiation (*λ* = 0.071073 nm). Data for **P2a** and **P2b** were reported previously.^35^

The above results revealed that **P4a** and **P4b** have a regular backbone structure with exact repeat units, while all the polymers (**P1–P3**) synthesized *via* the Stille coupling polymerization contain significant amounts of structural defects. Among the Pd catalyst systems used, Pd(PPh_3_)_4_ afforded the most regular backbone architecture observed in **P3**, while Pd(PPh_3_)_2_Cl_2_ produced the most irregular polymer backbone in **P1**. One would intuitively expect that the molecular ordering of these polymers in the solid state would follow the order of **P4** > **P3** > **P2** > **P1** based on their structural regularity. We investigated the chain packing of these polymers in spin-coated thin films using X-ray diffractometry (XRD). As shown in Fig. 4, **P1–P3** films are highly crystalline with prominent primary (100) and secondary (200) diffraction peaks that represent the typical layer-by-layer lamellar polymer chain packing.^5^ The absence of the (010) peak that represents the π–π stacking distance (at 2*θ* = ∼20–25°) indicates that the polymer chains adopted an edge-on orientation.^5^ As anticipated, the crystallinity of the **P1–P3** films follows the same order of **P3** > **P2a** > **P1** (having the same C20 side chains) as that of their structural regularity, judging from the intensities of their diffraction peaks. Much to our surprise, the structurally regular polymer **P4a** showed extremely low diffraction intensities, suggesting the very low crystallinity of this polymer. **P4b**, which has C24 side chains, also showed much poorer molecular ordering compared to **P2b** that has the same C24 side chains. The reason for the unexpected poor crystallinity of **P4a** and **P4b** is not fully understood at this time and needs to be further investigated. We suspect that the regular backbone structure of these two polymers would result in a high degree of rigidity for the polymer main chain, hampering the packing of the polymer molecules. On the other hand, the structural defects present in **P1–P3** would provide a desirable flexibility to the polymer backbone, allowing for a slower crystallization process and a fine tuning of the chain packing. Transmission XRD measurements were conducted on polymer flakes to determine the π–π stacking distance between polymer backbones (Fig. 5). All polymers showed distinct (010) reflection peaks around 2*θ* = 10.6–10.8°, which correspond to π–π stacking distances of 0.38–0.39 nm (Table 2). These results suggest that the existence of the structural defects in **P1–P3** did not alter the π–π stacking distance noticeably and would probably not markedly influence the hopping of charge carriers along the π–π stacks.

**Table 2:** Summary of XRD results and OTFT performance of polymers **P1–P4**^*a*^

Polymer	Anneal. temp. (°C)	XRD		OTFT performance		
		*d*_(100)_ (nm)	*d*_(010)_ (nm)	Ave. (max) *µ*_h_ (cm^2^ V^–1^ s^–1^)	*I*_on_/*I*_off_	*V*_th_ (V)
**P1**	150	1.91		1.60 (2.10)	∼10^6^	–6
	200	1.92	0.38	2.84 (2.94)	∼10^4^	–3
**P2a**	150	1.91		1.58 (2.10)	∼10^4^	–10
	200	1.88	0.38	3.57 (5.50)	∼10^6^	–7
**P2b**	150	2.13		2.65 (3.37)	∼10^4^	18
	200	2.11	0.39	1.46 (1.51)	∼10^4^	18
**P3**	150	1.91		1.62 (1.74)	∼10^4^	17
	200	1.89	0.38	1.35 (1.41)	∼10^4^	19
**P4a**	150	1.98		0.065 (0.084)	∼10^8^	–22
	200	1.98	0.38	0.029 (0.036)	∼10^5^	–35
**P4b**	150	2.15		0.90 (0.94)	∼10^6^	–8
	200	2.13	0.39	1.10 (1.13)	∼10^6^	–24

^*a*^Data for **P2a** and **P2b** were reported previously.^34,35^

The crystallinity of these polymers was also studied by differential scanning calorimetry (DSC) (data are presented in the ESI†). The DSC results showed that for all the polymers having the C20 side chains (**P1**, **P2a**, **P3**, and **P4a**) only **P1** showed a melting point at 287 °C in the testing temperature range from –20 °C to 325 °C. **P2a** and **P3** may have melting points higher than their decomposition temperatures, while **P4a** probably does not have an obvious melting point based on its poor crystallinity determined by the thin film XRD. For **P2b** and **P4b**, which have C24 side chains, **P2b** showed a distinct melting point at 293 °C, while there was no thermal transition observed for **P4b**. The melting point of **P4b** may also be above its thermal decomposition temperature. Obviously, the existence of structural defects could lower the melting points of the polymers as observed in **P1** and **P2b**. The structural defects might have helped the creep of the polymer chains in achieving more ordered chain packing.

**Fig. 6:**
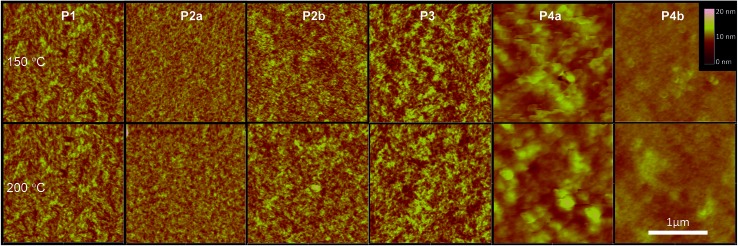
AFM height images (2 µm × 2 µm each) of **P1–P4** thin films (∼50 nm) spin-coated on dodecyltrichlorosilane-modified Si/SiO_2_ wafers and annealed at 150 °C or 200 °C. Images of **P2b** were reported previously.^35^

Interconnections of grains in polymer thin films are crucial for charge transport in polymer semiconductors. The atomic force microscopy (AFM) images of polymers **P1**, **P2**, and **P3** exhibited similar surface morphologies composed of well-connected small grains (Fig. 6). The **P2a** and **P2b** films are very smooth followed by an increase in roughness for **P1**. **P3** has the roughest surface among the four polymers synthesized by the Stille coupling polymerization. **P4a** and **P4b** showed dramatically different morphologies compared with **P1–P3**. **P4a** films showed a very rough morphology comprising of large flakes ∼100 nm in size with the films of **P4b** being quite smooth composed of small donut-shaped structures.

**Fig. 7:**
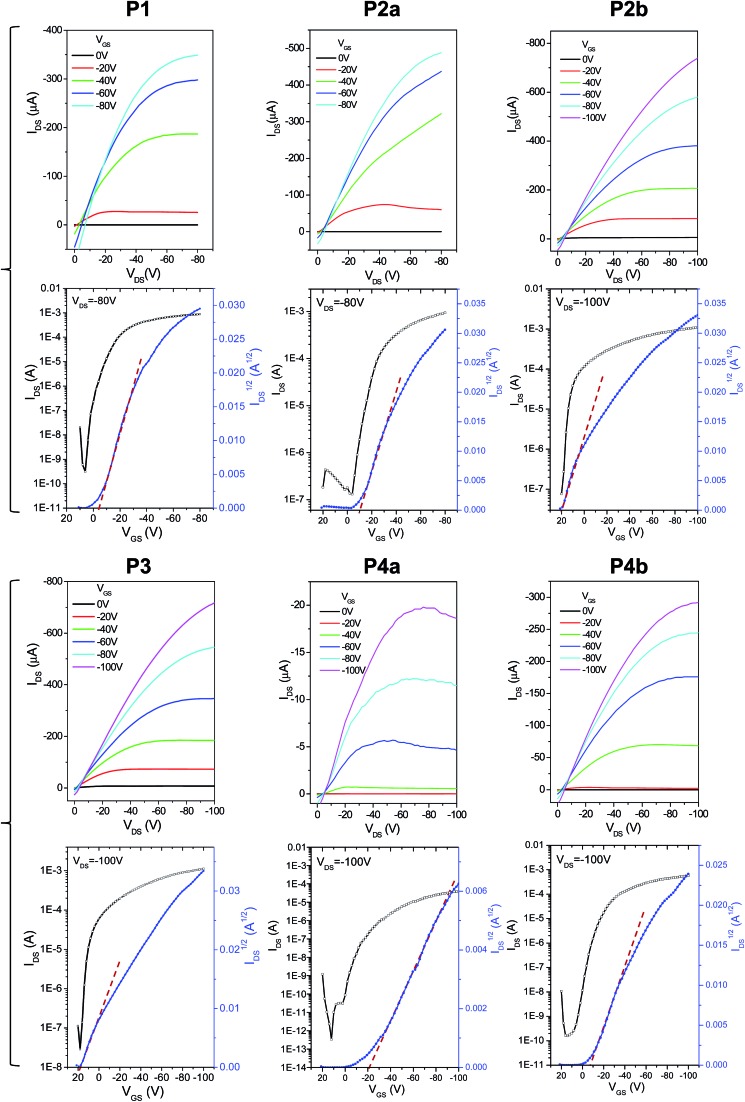
Output (top) and transfer (bottom) characteristics of typical bottom-gate bottom-contact OTFT devices using polymers **P1–P4** as channel semiconductors. The polymer thin films were annealed at 150 °C for 15 min. Device dimensions: *L* = 30 µm; *W* = 1 mm. N-doped Si/SiO_2_ wafers were used as substrates. The thickness of the dielectric SiO_2_ layer is 200 nm for **P1** and **P2a** and 300 nm for the other polymers. Curves for **P2a** and **P2b** were reported previously.^35^

The charge transport performance of these polymers was evaluated in bottom-gate bottom-contact OTFT devices fabricated on heavily n-doped Si/SiO_2_ wafers, where the Si layer was used as the gate electrode, the SiO_2_ layer as the dielectric, and gold pairs as source and drain electrodes. Output and transfer curves of typical OTFT devices are provided in Fig. 7. It can be seen in Table 2 that the 150 °C-annealed thin films of **P1**, **P2a**, and **P3** with the same C20 side chains showed very similar hole mobilities with average values of ∼1.6 cm^2^ V^–1^ s^–1^. When the polymer films were annealed at 200 °C, the average mobility for **P1** and **P2a** increased to 2.84 cm^2^ V^–1^ s^–1^ and 3.57 cm^2^ V^–1^ s^–1^, respectively. On the other hand, the average mobility for the 200 °C-annealed **P3** dropped slightly to 1.35 cm^2^ V^–1^ s^–1^. **P4a** having the same C20 side chains showed strikingly lower mobilities of 0.065 and 0.029 cm^2^ V^–1^ s^–1^ for the 150 °C- and 200 °C-annealed thin films, respectively, which is most likely due to the very poor crystallinity (Fig. 4) and the poor intergranular connections (Fig. 6) of the polymer films. It was also noted that the mobilities of **P4b** (150 °C-annealed: 0.90 cm^2^ V^–1^ s^–1^; 200 °C-annealed: 1.10 cm^2^ V^–1^ s^–1^) are much lower than those of **P2b** having the same C24 side chains (150 °C-annealed: 2.65 cm^2^ V^–1^ s^–1^; 200 °C-annealed: 1.46 cm^2^ V^–1^ s^–1^), which can also be attributed to the poorer crystallinity of **P4b**. Some devices showed curved *I*_DS_^1/2^*vs. V*_GS_ lines (Fig. 4) and thus gate-dependent mobilities (ESI†). This bending phenomenon was also observed previously for PDQT^25,34,35^ and other high mobility polymers,^27,29,42,43^ which was explained by the carrier supersaturation in the conduction channel at high source/drain current regimes or low-density shallow charge trapping in the semiconductor and/or interface.^27^

Based on the above results, it appears that the presence of an appropriate amount of structural defects in the polymers synthesized by the Stille coupling polymerization could provide good solubility, increase molecular ordering, maintain short π–π stacking distance, and improve film morphology. These characteristics favorably result in the enhanced charge transport performance of these polymers in OTFTs. The structurally regular polymers **P4a** and **P4b** showed very poor solution processability (poor solubility), low crystallinity, and poor morphology, leading to much lower charge carrier mobility in comparison to the “defected” polymers **P1–P3**.

## Conclusions

We analyzed the structures of PDQT polymers synthesized using different polymerization methods and observed a correlation between the structure and the optoelectronic properties of these polymers. Our results confirmed the formation of the DBT–DBT and BT–BT structural defects due to the homocoupling side reactions of the C–Br bonds and the organostannane species, respectively, during the Stille coupling polymerization in the presence of three different Pd catalysts. We found that Pd(PPh_3_)_4_ produced a PDQT polymer with fewest structural defects, while Pd(PPh_3_)_2_Cl_2_ gave the most irregular polymer backbone architecture. NMR measurements of the polymers at an elevated temperature of 125 °C revealed peaks representing the structural defects with an aid of the NMR spectra of the structurally well-defined polymers prepared by Yamamoto coupling with Ni(COD)_2_. MALDI-ToF mass spectroscopy further substantiated the presence of the DBT–DBT and BT–BT structural defects formed during the Stille coupling polymerization by detecting chains containing comonomer ratios not equal to the expected theoretical values. Isolation of a by-product from model Stille coupling reactions using these Pd catalyst systems validated that homocoupling involving the C–Br bonds occurred to a significant extent during the Stille coupling reactions, which is responsible for the formation of the DBT–DBT structural defects in the polymers. The existence of these structural defects was thought to result in the redshifts of the absorption profiles of the PDQT polymers. Surprisingly, the “perfect” structurally well-defined PDQT polymers prepared using Yamamoto coupling showed very poor molecular ordering in thin films presumably due to their rigid backbone that prevents the polymer chains from fine adjustment to form highly ordered molecular packing. As a result, these “perfect” polymers showed poor charge transport performance in OTFTs. On the contrary, polymers having significant amounts of structural defects prepared by the Stille coupling polymerization exhibited much improved molecular ordering and OTFT performance. Our results demonstrated that a “perfect” regular polymer may not lead to the best OTFT performance, while the existence of an appropriate/acceptable amount of structural defects in polymer chains can enhance the molecular ordering and thus the charge transport.

## Supplementary Material

Supplementary information
