# Supplementary material for: Is a polymer semiconductor having a “perfect” regular structure desirable for organic thin film transistors?
Source: Chem Sci. 2015 Mar 31;6(5):3225–35. doi: 10.1039/c5sc00843c (PMC5657407; doi:10.1039/c5sc00843c)
Supplement: Supplementary file 1 [file SC-006-C5SC00843C-s001.pdf]

Electronic Supplementary Material (ESI) for Chemical Science.
This journal is © The Royal Society of Chemistry 2015

Supplementary Information

Is a polymer semiconductor having a "perfect" regular structure desirable for organic thin film transistors?

Wei Hong,[a] Shaoyun Chen,[abd] Bin Sun,[a] Mark A. Arnould,[c] Yuezhong Meng,[b] Yuning Li*[a]

[a] Department of Chemical Engineering and Waterloo Institute for nanotechnology (WIN), University of Waterloo, 200 University Avenue West, Waterloo, Ontario, Canada N2L 3G1; [b] The Key Laboratory of Low-carbon Chemistry & Energy Conservation of Guangdong Province / State Key Laboratory of Optoelectronic Materials and Technologies, Sun Yat-Sen University, Guangzhou 510275, P. R. China; [c] Xerox Corporation, 800 Phillips Road, Bldg. 0139-64A, Webster, NY 14580, USA

[d] Current address: College of Chemistry and Life Science, Quanzhou Normal University, Quanzhou 362000, Fujian, P.R. China

1. Instrumentation and materials.

^{1}H NMR spectra of polymers were acquired in duterated 1,1,2,2-tetrachloroethane (TCE)-d_2 using a Bruker Advance 500 at room temperature and 125 °C. ^{1}H and ^{13}C NMR of other compounds were acquired on a Bruker DPX 300 MHz spectrometer at room temperature. A Polymer Labs PL-GPC 220 high-temperature gel-permeation chromatography (GPC) system was used to determine the molecular weight of the polymers using 1,2,4-trichlorobenzene (TCB) as an eluent with polystyrene standards and a column temperature of 140 °C. MALDI-ToF measurements were conducted using a Bruker Ultraflex II ToF/ToF mass spectrometer in reflector mode after GPC separation and spray deposition (from chloroform solutions) onto a target with all-*trans*-retinoic acid as the matrix. UV-Vis spectra were acquired using a Thermo Scientific Genesys 10 UV spectrometer with a polymer solution in 1,1,2,2-tetrachloroethane (TCE) or a thin film on quartz glass. Reflective XRD spectra of polymer thin films (~50 nm) spin-coated on SiO_2/Si substrates were obtained with a Bruker D8 Advance powder

diffractometer with Cu Kα1 radiation (λ = 1.5406 Å). Transmission XRD measurements of polymer flakes between two Mylar substrates were carried out using a Bruker Smart Apex2 CCD with Mo Kα radiation (λ = 0.71073 Å). The atomic force microscopy (AFM) images of polymer thin films (~50 nm) spin-coated on SiO_2/Si substrates were measured using a Dimension 3100 Scanning Probe Microscope. A CHI600E Electrochemical Analyzer was used for the cyclic voltammetry (CV) measurements of polymer thin films in a 0.1 M tetrabutylammonium hexafluorophosphate solution in anhydrous acetonitrile at a scan rate of 50 mV s^{-1}. Ferrocene was used as an external reference, which has a highest occupied molecular orbital (HOMO) energy level of -4.8 eV.[1] Bottom-gate bottom-contact (BGBC) organic thin-film transistor (OTFT) configuration was used for the evaluation of polymer semiconductors. Heavily n-doped silicon wafers having a thermally grown SiO_2 layer with a thickness of 200 nm (capacitance, C_i = ~17 nF·cm^{-2}) (for **P1** and **P2a**) or 300 nm (C_i = ~11 nFcm^{-2}) (for **P2b**, **P3**, **P4a**, and **P4b**) were used as the substrate, where Si functioned as the gate and SiO_2 as the dielectric. Gold source and drain pairs (channel width, L = 1 mm; channel length, W = 30 μm) were patterned using a common photolithography technique. The polymer semiconductor layer was deposited on the dodecyltrichlorosilane-modified SiO_2/Si substrate by spin-coating a polymer solution in TCE and 1,2-dichlorobenzene (ODCB) (v/v = 1/1). The resulting polymer thin film (~35 nm) was annealed at 150 °C or 200 °C for 15 min in a glove box. The device was encapsulated with a ~500 nm PMMA layer before testing in the air. The more detailed device fabrication and characterization procedures were reported previously.[2]

Monomer **M1a** [2a] and **M1b** [2b] were synthesized following the procedures reported previously. Synthesis and characterization of polymer **2a** [3] and **2b** [2b] were reported previously. Other chemicals were obtained from commercial sources and used as received.

2. Synthetic Procedures

2.1. Synthesis of polymer P1

P1 was synthesized following a previously reported procedure.[4]

2,5-Bis(2-octyldodecyl)-3,6-di(thiophen-2-yl)pyrrolo[3,4-*c*]pyrrole-1,4(2*H*,5*H*)-dione **M1a** (0.6115 g, 0.6 mmol) and 5,5'-bis(trimethylstannyl)bithiophene (0.2951 g, 0.6 mmol) were charged to a dry 100 mL-flask. After degassing and refilling with argon 3 times, anhydrous toluene (40 mL) and bis(triphenylphosphine)palladium(II) dichloride ($Pd(PPh_3)_2Cl_2$) (14 mg, 0.02 mmol) were added. The reaction mixture was heated to 90 °C and stirred for 48 hr. Bromobenzene (0.5 mL) was added to the reaction mixture and then stirred under reflux for 3 h before cooling down to room temperature. The final mixture was poured into stirring ethanol (400 mL) and the resultant solid was filtered off, washed with methanol and dried. The solid was then further purified by Soxhlet extraction using acetone, hexane and finally dissolved with chloroform. Yield: 600 mg (97.7%).

Data for **P1** follow. HT-GPC: M_n = 21.4 kg/mol; PDI = 2.03. UV-Vis: λ_{max} = 775 nm (in TEC); 783 nm (film).

2.2. Synthesis of polymer P3

2,5-Bis(2-octyldodecyl)-3,6-di(thiophen-2-yl)pyrrolo[3,4-c]pyrrole-1,4(2*H*,5*H*)-dione **M1a** (0.3058 g, 0.3 mmol) and 5,5'-bis(trimethylstannyl)bithiophene (0.1476 g, 0.3 mmol) were charged to a dry 50 mL-flask. After degassing and refilling with argon 3 times, tetrakis(triphenylphosphine)palladium(0) ($Pd(PPh_3)_4$) (13.9 mg, 0.012 mmol) and anhydrous toluene (15 ml) were added under an argon atmosphere. The mixture was stirred for 48 h at 110 ºC. After cooling to room temperature, the reaction mixture was poured into a stirring methanol (100 ml) and stirred for 1 h and then filtered to give a solid.

The solid was further purified by Soxhlet extraction using acetone, hexane and then dissolved with chloroform. The chloroform extraction was dried in vacuo to produce polymer **P3**. Yield: 303 mg (98.7%).

Data for **P3** follow. HT-GPC: M_n = 38.3 kg/mol; PDI = 2.16. UV-Vis: λ_{max} = 798 nm (in TEC); 787 nm (film).

2.3. Synthesis of polymer P4

Polymers **P4a** and **P4b** were synthesized according to the route outlined in Scheme S1.

Scheme S1. The synthetic route to monomers **M2** and polymers **P4**.

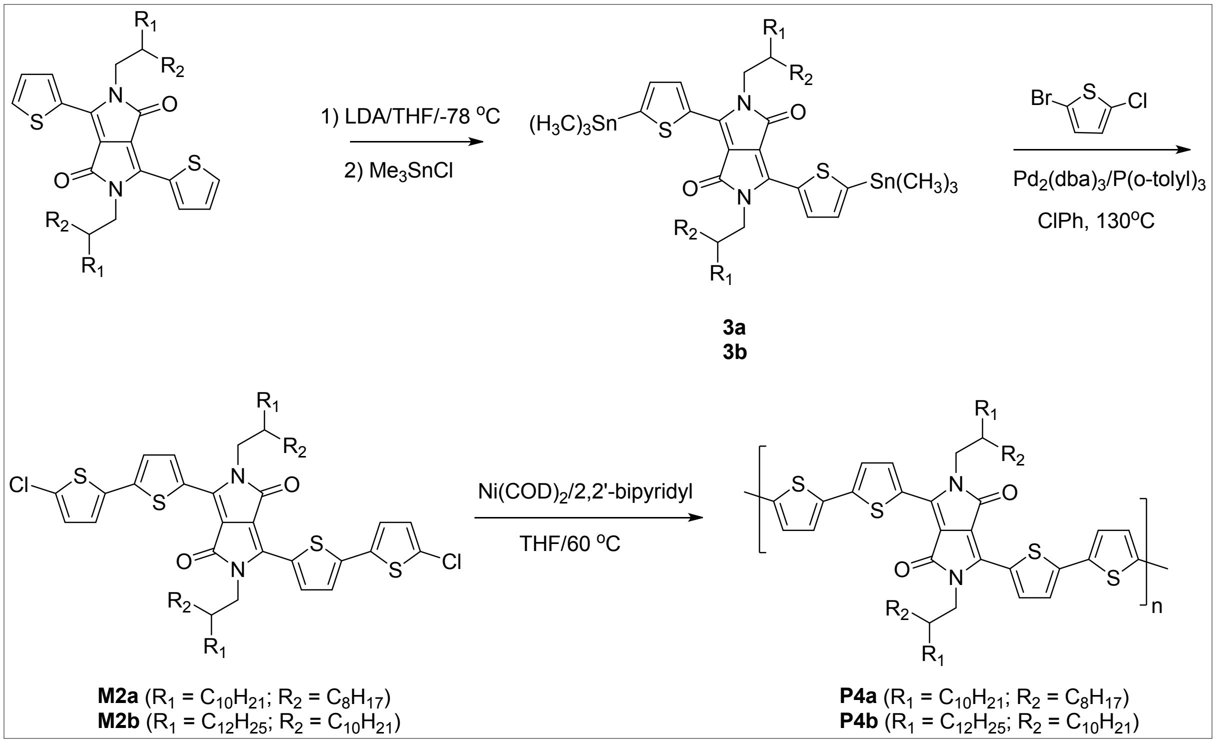

2,5-Bis(2-octyldodecyl)-3,6-bis(5-(trimethylstannyl)thiophen-2-yl)pyrrolo[3,4-*c*]pyrrole-1,4(2*H*,5*H*)-dione (3a)

The synthesis of this compound was reported previously. [5]

2,5-Bis(2-octyldodecyl)-3,6-di(thiophen-2-yl)pyrrolo[3,4-*c*]pyrrole-1,4(2*H*,5*H*)-dione (1.033 g, 1.2 mmol) was dissolved in 40 mL of anhydrous THF under argon. The solution was cooled to -78 °C and lithium diisopropylamine (LDA) (1.8 mL, 3.6 mmol, 2 M in hexane) was added drop-wise. The reaction mixture was stirred at this temperature for 1 h and allowed to warm to -20-0 °C and stirred for an additional 1 h. The mixture was then cooled to -78 °C and trimethyltin chloride (4.2 mL, 4.2 mmol, 1 M in THF) was added rapidly. The mixture was allowed to warm to room temperature and stirred overnight. The reaction was quenched with 30 mL of ice-water and extracted with CH_2Cl_2 three times. The organic extraction was washed with de-ionized (DI) water twice and then dried over anhydrous Na_2SO_4. After removal of the solvent in vacuo, the residue was washed by methanol to give compound **3a** (1.40 g, 98.3%).

^{1}H-NMR ($CDCl_3$, 300 MHz, ppm) δ 0.44 (s, 18H); 0.86 (t, 12H); 1.21-1.29 (m, 64H); 1.88 (m, 2H); 4.01-4.03 (d, 4H); 7.31(d, 2H, J = 3Hz), 8.95(d, 2H, J = 3Hz).

3,6-Bis(5'-chloro-2,2'-bithiophen-5-yl)-2,5-bis(2-octyldodecyl)pyrrolo[3,4-*c*]pyrrole-1,4(2*H*,5*H*)-dione (M2a)

Compound **3a** (0.5587g, 0.47 mmol) was added to a dry 50 mL-flask. After degassing and refilling with argon 3 times, 2-bromo-5-chlorothiophene (0.1949 g, 0.987 mmol) and anhydrous toluene (35 ml) was added under argon. Then $Pd(PPh_3)_2Cl_2$ (7 mg, 0.01 mmol) was added to the solution and the mixture was stirred at 100 °C under argon for 48 h. After removal of solvent in vacuo, the residue was purified

by column chromatography on silica gel (hexane : DCM = 2 : 1), followed by recrystallization from isopropanol three times, to produce **M2a** (0.11g, 20.1 %).

^{1}H-NMR ($CDCl_3$, 300 MHz, ppm) δ 0.86 (t, 12H); 1.21-1.29 (m, 64H); 1.92-1.93(m, 2H); 4.00-4.02 (d, 4H), 6.88 (d, 2H, J = 3Hz), 7.07(d, 2H, J = 3Hz), 7.22 (d, 2H, J = 3Hz), 8.85(d, 2H, J = 3Hz). ^{13}C NMR (CDCl3, 75 MHz, ppm): δ 161.56, 141.50, 139.39, 136.44, 134.80, 130.80, 128.39, 127.34, 124.73, 124.19, 108.55, 46.30, 37.93, 31.89, 31.32, 30.04, 29.65, 29.55, 29.37, 29.31, 26.34, 22.67, 14.11. HRMS (ESI, $[M+H]^+$): found 1093.5297, calcd. 1093.5340.

Poly(3,6-bis(2,2'-bithiophen-5-yl)-2,5-bis(2-octyldodecyl)pyrrolo[3,4-*c*]pyrrole-1,4(2*H*,5*H*)-dione) (P4a)

2,2'-Bipyridyl (16 mg, 0.104 mmol), **M2a** (0.095g, 0.088 mmol), and anhydrous THF (10 mL) were added to a dry 50 mL-flask under argon followed by the addition of bis(1,5-cyclooctadiene)nickel(0) ($Ni(COD)_2$) (0.0286 g, 0.04 mmol) while keeping the mixture under argon protection. The mixture was stirred at room temperature for 15 min and then at 60 °C for 24 h. The mixture was cooled and poured into 100 mL of stirring methanol and the final product was filtered and collected. The solid was then further purified by Soxhlet extraction using acetone for 4 h, hexane for 12 h, and chloroform for 6 h. The residue was further extracted with TCE for 12 h yielding the product (31 mg, 34.0%).

2,5-Bis(2-decyltetradecyl)-3,6-bis(5-(trimethylstannyl)thiophen-2-yl)pyrrolo[3,4-*c*]pyrrole-1,4(2*H*,5*H*)-dione (3b)

2,5-Bis(2-decyltetradecyl)-3,6-di(thiophen-2-yl)pyrrolo[3,4-c]pyrrole-1,4(2*H*,5*H*)-dione (1.266 g, 1.3 mmol) was dissolved in anhydrous THF (45 mL) under argon. The solution was cooled to -78 °C and

LDA (1.95 mL, 3.9 mmol, 2 M in hexane) was added drop-wise. The reaction mixture was stirred at this temperature for 1 h then allowed to warm to -20-0 °C and stirred for an additional 1 h. The mixture was cooled back to -78 °C and trimethyltin chloride (4.55 mL, 4.55 mmol, 1M in THF) was added rapidly. The final mixture was allowed to warm to room temperature and stirred overnight. The reaction was quenched with 30 mL of ice-water and extracted with CH_2Cl_2 three times. The organic extraction was washed with DI water twice and then dried over anhydrous Na_2SO_4. After removal of the solvent in vacuo, the residue was washed by methanol to produce compound **3b** (1.54 g, 91.1%).

^{1}H-NMR ($CDCl_3$, 300 MHz, ppm) δ 0.44 (s, 18H); 0.70-0.87 (m, 12H); 1.20-1.40 (m, 80H), 1.90(m, 2H); 4.02-4.05 (d, 4H); 7.21(d, 2H, *J* = 3Hz), 8.95(d, 2H, *J* = 3Hz).

3,6-Bis(5'-chloro-[2,2'-bithiophen]-5-yl)-2,5-bis(2-decyltetradecyl)pyrrolo[3,4-*c*]pyrrole-1,4(2*H*,5*H*)-dione (M2b)

Compound **3b** (0.6496 g, 0.5 mmol) was added to a dry 50 mL-flask. After degassing and refilling with argon 3 times, 2-bromo-5-chlorothiophene (0.2074g, 1.05 mmol) and anhydrous chlorobenzene (40 ml) were added under argon atmosphere followed by the addition of $Pd(PPh_3)_2Cl_2$ (7 mg, 0.01 mmol). The mixture was stirred for 48 h at 130 °C then the solvent was removed in vacuo The residue was separated on a silica gel column (hexane : DCM = 2 : 1). The purified solid was recrystallized from isopropyl three times to yield compound **M2b** (0.34 g, 56.4% yield).

^{1}H-NMR ($CDCl_3$, 300 MHz, ppm) δ 0.83-0.88 (t, 12H); 1.21-1.29 (m, 80H); 11.92(m, 2H); 4.00-4.02 (d, 4H), 6.88-6.89(d, 2H, *J* = 3Hz), 7.07-7.08 (d, 2H, *J* = 3Hz), 7.22-7.23 (d,2H, *J* = 3Hz), 8.85-8.87(d, 2H, *J* = 6Hz). ^{13}C NMR (CDCl3, 75 MHz, ppm): δ 161.51, 141.48, 139.33, 136.46, 134.81, 130.79,

128.39, 127.33, 124.71, 124.15, 108.52, 46.30, 37.93, 31.93, 31.34, 30.05, 29.68, 29.62, 29.38, 26.35, 22.70, 14.11. HRMS (ESI, $[M+H]^+$): found 1205.6543, calcd. 1205.6592.

Poly(2,5-bis(2-decyltetradecyl)-3,6-bis(5'-methyl-[2,2'-bithiophen]-5-yl)pyrrolo[3,4-*c*]pyrrole-1,4(2*H*,5*H*)-dione) (P4b)

2,2'-Bipyridyl (20 mg, 0.13 mmol), **M2b** (0.150g, 0.124 mmol) and anhydrous THF (12 mL) were placed in a dry 50 mL flask. $Ni(COD)_2$ (36 mg, 0.13 mmol) was added to the mixture under argon. The mixture was stirred at room temperature for 15 min and at 60 °C for 24 h. The reaction mixture was cooled to room temperature and poured into 100 mL of stirring methanol. The resultant solid was filtered and further purified by Soxhlet extraction using acetone for 4 h and hexane for 12 h. This residue was extracted with chloroform for 6 h giving 90 mg (yield 62.1%) of product and further extracted with TCE for 12 h giving an additional 36 mg (yield 24.8%) of the product. The HT-GPC (in 1,2,4-trichlorobenzene at 140 °C) data of the fraction extracted with chloroform: M_n = 23.8 kg/mol; M_w = 59.7 kg/mol; M_w/M_n = 2.51. The fraction extracted with chloroform was used for the characterization of this polymer in this study.

2.4 Model Stile coupling reactions

The general synthetic procedure for model compounds 3,6-di((2,2'-bithiophen)-5-yl)-2,5-bis(2-octyldodecyl)pyrrolo[3,4-*c*]pyrrole-1,4(2*H*,5*H*)-dione (1a) and 6,6'-((2,2'-bithiophene)-5,5'-diyl)bis(3-((2,2'-bithiophen)-5-yl)-2,5-bis(2-octyldodecyl)pyrrolo[3,4-*c*]pyrrole-1,4(2*H*,5*H*)-dione) (2a)

Scheme S2. (This scheme is same as Scheme 2 shown in the main text.) a) Model Stille coupling reactions using different catalyst systems: i) $Pd(PPh_3)_2Cl_2$/toluene/reflux; ii) $Pd_2(dba)_3$/$P(o\text{-}tol)_3$ (molar ratio: 1/4)/chlorobenzene/130 °C; iii) $Pd(PPh_3)_4$/toluene/reflux. b) Proposed formation of structural defects DBT-DBT and BT-BT via the homocoupling side reactions.

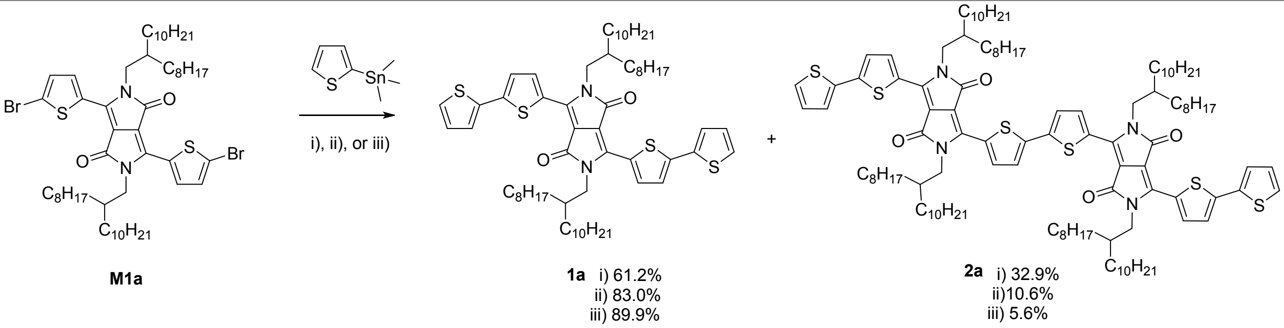

3,6-Bis(5-bromothiophen-2-yl)-2,5-bis(2-octyldodecyl)pyrrolo[3,4-*c*]pyrrole-1,4(2*H*,5*H*)-dione (**M1a**) (1.0 equiv.) and 2-trimethylstannylthiophene (2.1 equiv.) were added to a dry 25 mL-flask. After degassing and refilling with argon 3 times, dry solvent (5 ml) and catalyst were added under an argon atmosphere. The mixture was heated for 48 h. The reaction conditions for the three catalyst systems are summarized below.

Condition	Catalyst system (mol% based on **M1a**)	Solvent	Temperature, °C
i	*Trans*-dichlorobis(triphenylphosphine) palladium(II) ($Pd(PPh_3)_2Cl_2$) (4 mol %)	Toluene	110
ii	Tris(dibenzylideneacetone)dipalladium(0) ($Pd_2(dba)_3$) (2 mol %) and tri(*o*-tol)phosphine ($P(o\text{-}tol)_3$) (8 mol %)	Chlorobenzene	130
iii	Tetrakis(triphenylphosphine) palladium(0) ($Pd(PPh_3)_4$) (4 mol %)	Toluene	110

After cooling to room temperature, the reaction mixture was separated by column chromatography on silica gel using an eluent of dichloromethane:hexane = 1:2~1:1, to give compounds **1a** and **2a**.

i. **1a** (52 mg, 61.2 %) and **2a** (28 mg, 32.9 %).

ii. **1a** (78 mg, 83.0 %) and **2a** (10 mg, 10.6 %).

iii. **1a** (80 mg, 89.9 %) and **2a** (5 mg, 5.6 %).

1a: ^{1}H-NMR ($CDCl_3$, 300 MHz, ppm) δ 0.83-0.88 (t, 12H); 0.95-1.50 (m, 64H); 1.97 (m, 2H); 4.02-4.05 (d, 4H), 7.06-7.09 (t, 2H, J = 4.2 Hz), 7.30-7.33 (m, 6H), 8.90-8.91 (d, 2H, J = 4.2 Hz). HRMS (ESI, M + H^+): found 1025. 6117, calcd. 1025.6120.

2a: ^{1}H-NMR ($CDCl_3$, 300 MHz, ppm) δ 0.82-0.86 (t, 24H); 0.95-1.50 (m, 128H); 1.96 (m, 4H); 4.03-4.05 (d, 8H, J = 4.2 Hz), 7.08-7.05 (t, 2H) 7.29-7.33 (m, 6H), 7.39-7.40 (d, 2H), 8.93-8.96 (t, 4H, J = 3.9 Hz). HRMS (ESI, M^+): found 1883.2202, calcd. 1883.2172.

3. Additional data

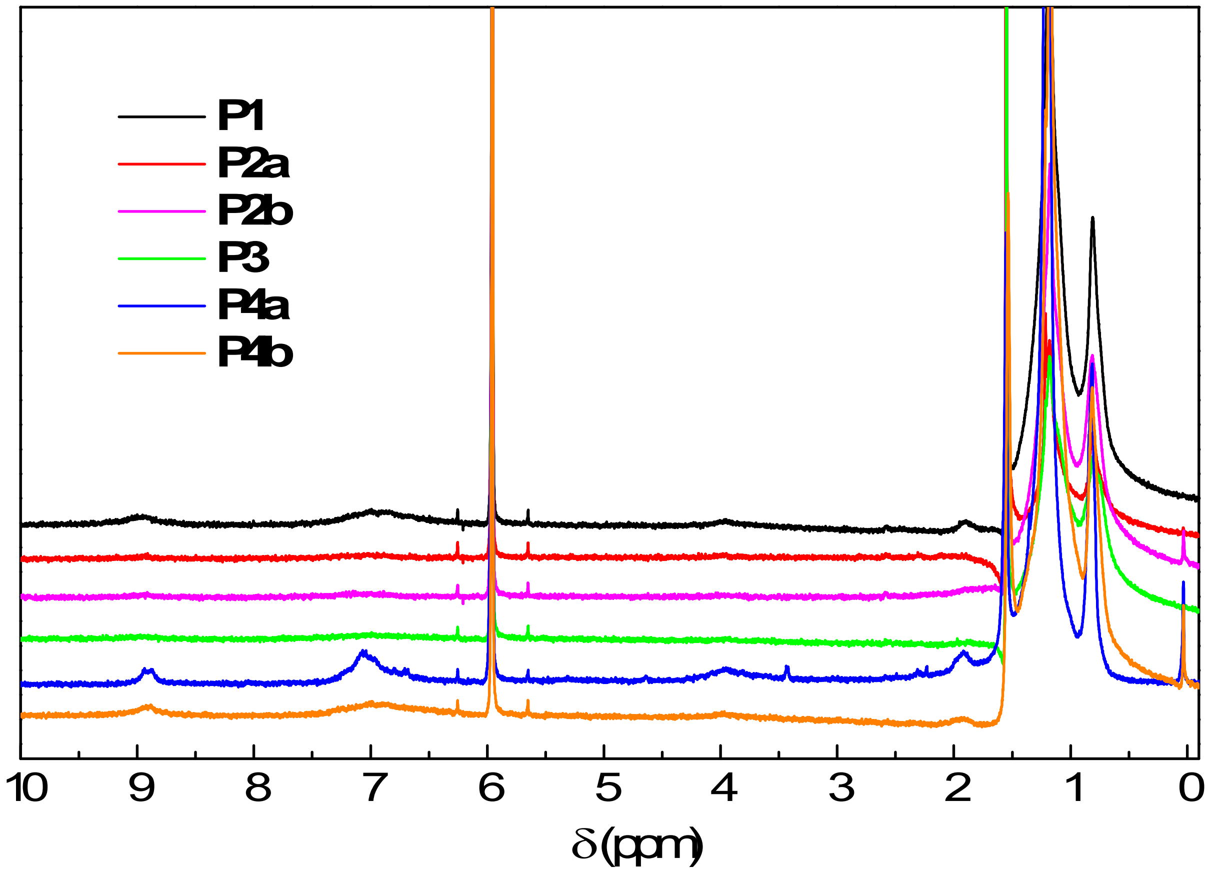

Fig. S1. 500 MHz ^{1}H NMR spectra of **P1**-**P4** acquired in duterated 1,1,2,2-tetrachloroethane (TCE-d_2) at room temperature.

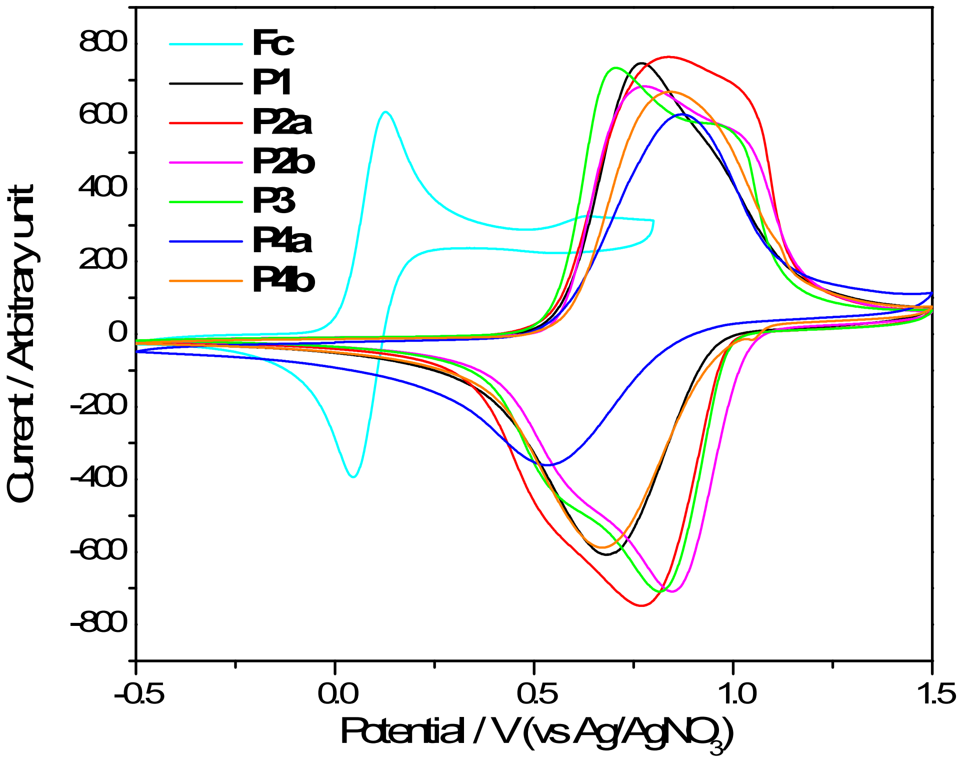

Fig. S2. Cyclic voltammograms (second cycles) of **P1**-**P4** thin films in 0.1 M tetrabutylammonium hexafluorophosphate in dry acetonitrile at a sweeping rate of 50 mV s^{-1} under nitrogen using ferrocene (Fc) as a reference.

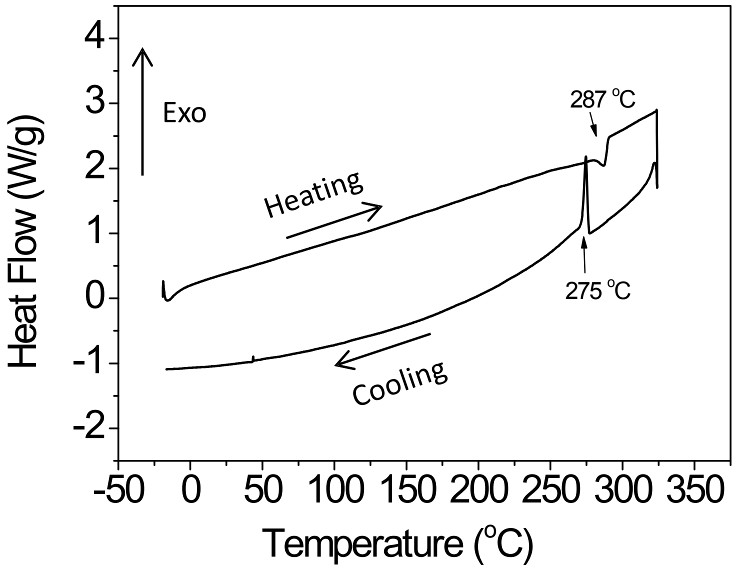

Fig. S3. DSC curve (second cycle) of **P1** measured with a heating rate of 10 °C min^{-1} under nitrogen. A positive heat flow indicates an exothermic (exo) event.

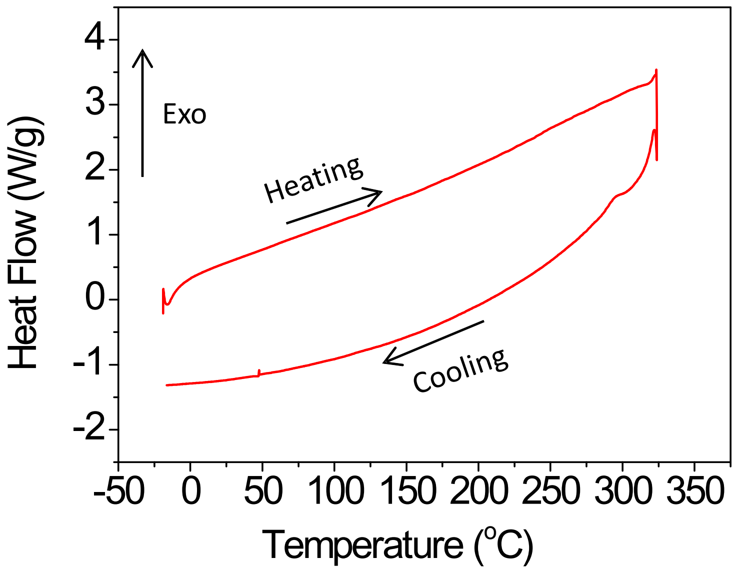

Fig. S4. DSC curve (second cycle) of **P2a** measured with a heating rate of 10 °C min^{-1} under nitrogen. A positive heat flow indicates an exothermic (exo) event.

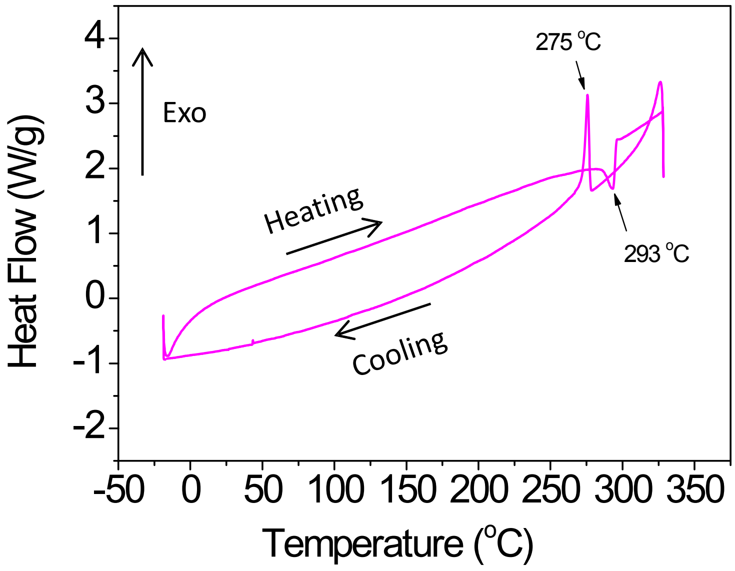

Fig. S5. DSC curve (second cycle) of **P2b** measured with a heating rate of 10 °C min^{-1} under nitrogen. A positive heat flow indicates an exothermic (exo) event.

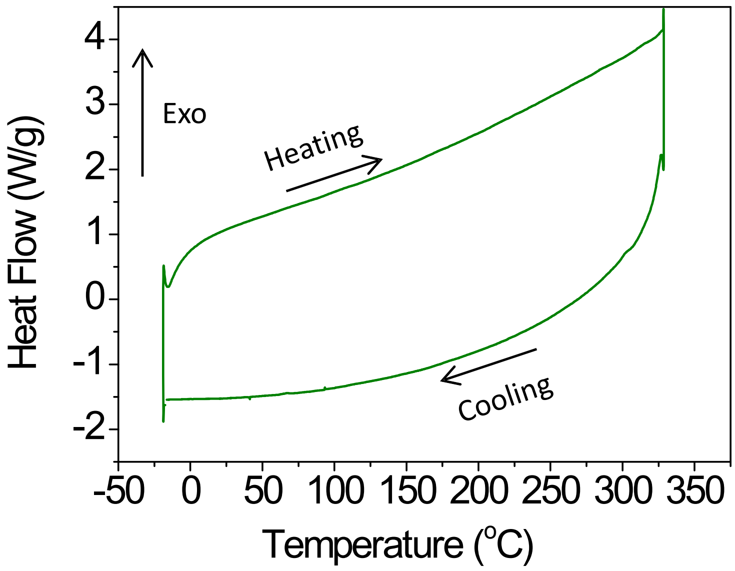

Fig. S6. DSC curve (second cycle) of **P3** measured with a heating rate of 10 °C min^{-1} under nitrogen. A positive heat flow indicates an exothermic (exo) event.

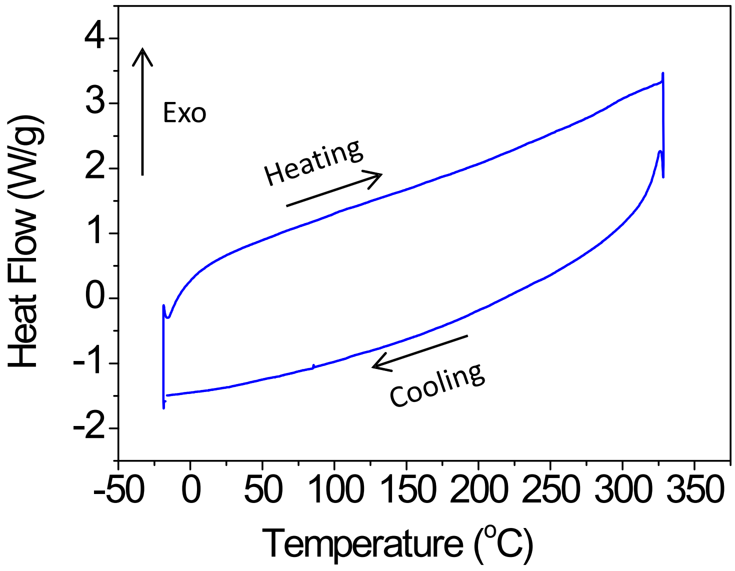

Fig. S7. DSC curve (second cycle) of **P4a** measured with a heating rate of 10 °C min^{-1} under nitrogen. A positive heat flow indicates an exothermic (exo) event.

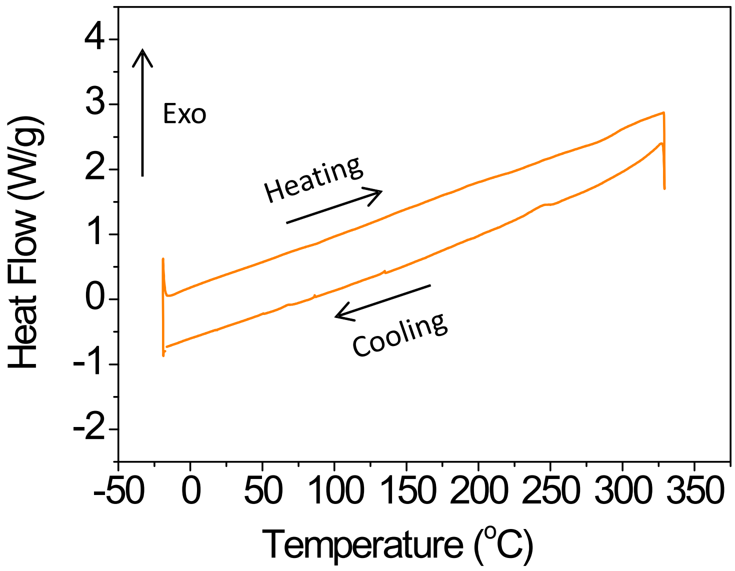

Fig. S8. DSC curve (second cycle) of **P4b** measured with a heating rate of 10 °C min^{-1} under nitrogen. A positive heat flow indicates an exothermic (exo) event.

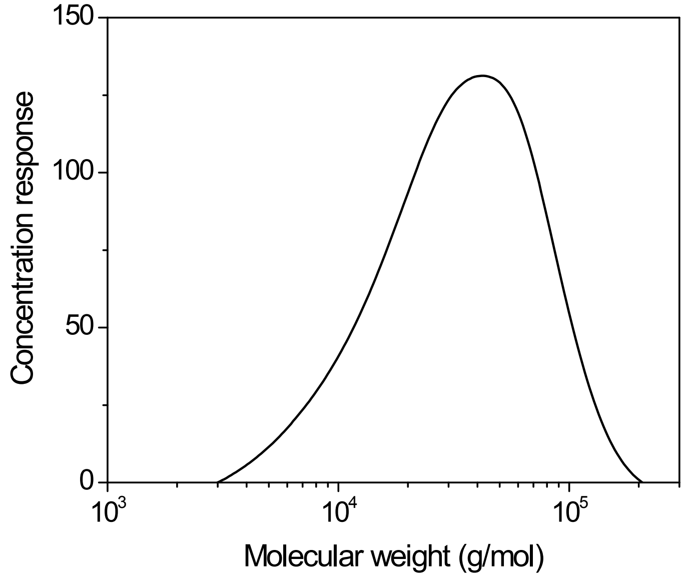

Fig. S9. Molecular weight distribution of **P1** measured by HT-GPC at 140 °C using 1,2,4-trichlorobenzne as eluent and polystyrene as standards. M_n = 21,400 g/mol; PDI = 2.03.

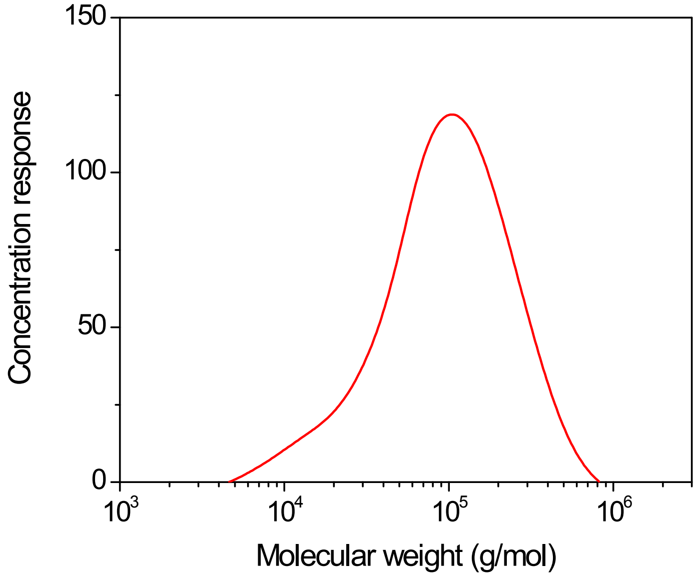

Fig. S10. Molecular weight distribution of **P2a** measured by HT-GPC at 140 °C using 1,2,4-trichlorobenzne as eluent and polystyrene as standards. M_n = 47,000 g/mol; PDI = 2.90.

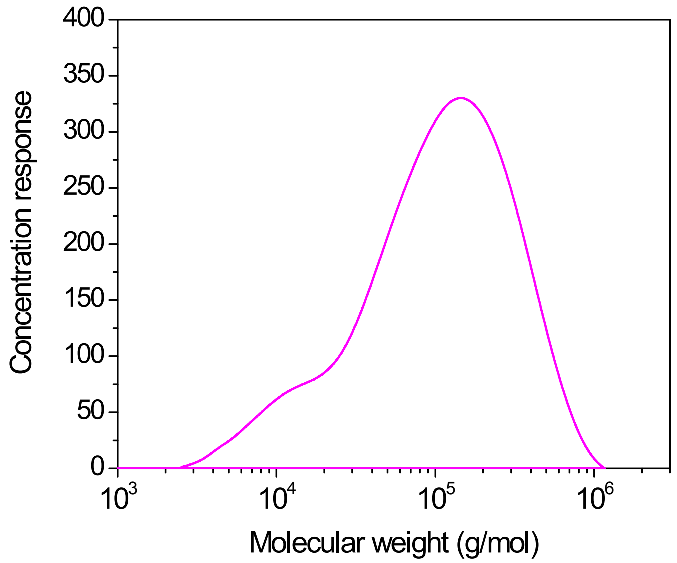

Fig. S11. Molecular weight distribution of **P2b** measured by HT-GPC at 140 °C using 1,2,4-trichlorobenzne as eluent and polystyrene as standards. M_n = 54,900 g/mol; PDI = 3.12.

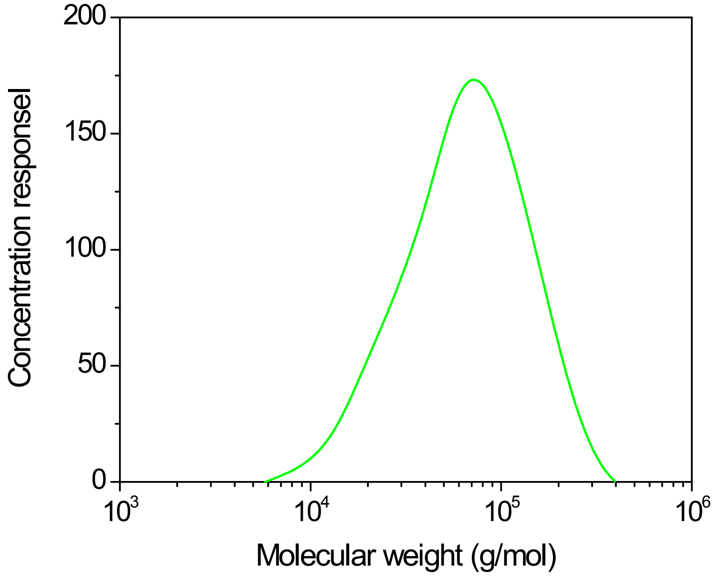

Fig. S12. Molecular weight distribution of **P3** measured by HT-GPC at 140 °C using 1,2,4-trichlorobenzne as eluent and polystyrene as standards. M_n = 38,300 g/mol; PDI = 2.16.

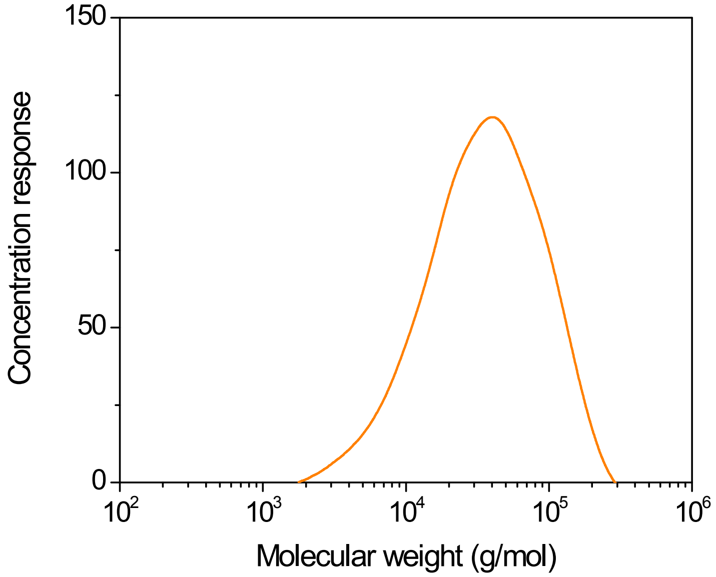

Fig. S13. Molecular weight distribution of **P4b** measured by HT-GPC at 140 °C using 1,2,4-trichlorobenzne as eluent and polystyrene as standards. M_n = 23,800 g/mol; PDI = 2.51.

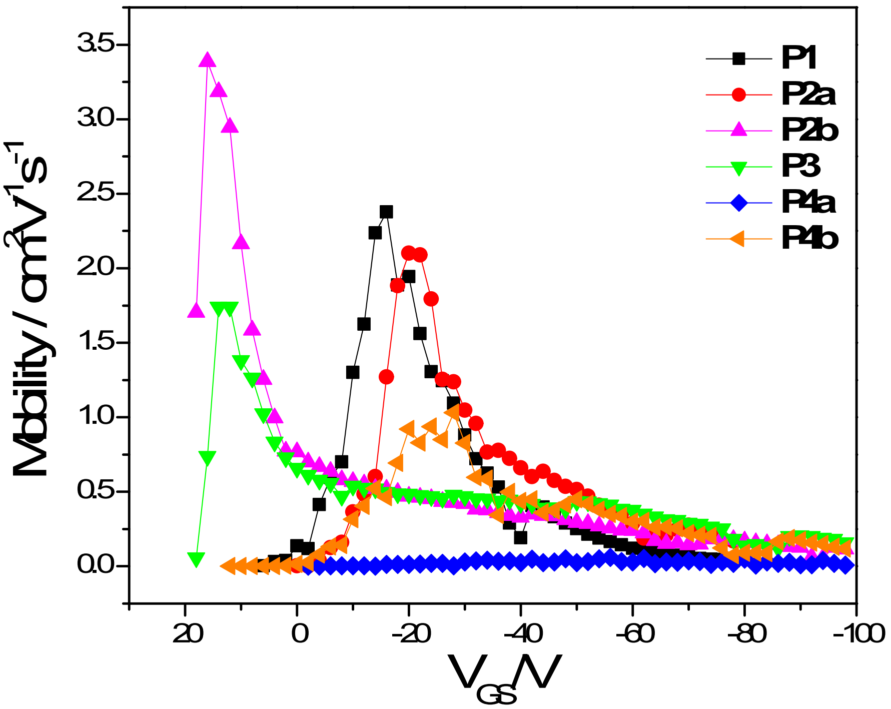

Fig. S14. Dependence of hole mobilities on the gate voltage extracted from the transfer curves of the OTFT devices shown in Fig. 7 in the main text.

References

1. B. W. D'Andrade, S. Datta, S. R. Forrest, P. Djurovich, E. Polikarpov and M. E. Thompson, *Org. Electron.,* 2005, **6**, 11-20.
2. (a) Y. Li, S. P. Singh and P. Sonar, *Adv. Mater.*, 2010, **22**, 4862-4866; (b) S. Chen, B. Sun, W. Hong, H. Aziz, Y. Meng and Y. Li, *J. Mater. Chem. C*, 2014, **2**, 2183-2190.
3. B. Sun, W. Hong, H. Aziz, N. M. Abukhdeir and Y. Li, *J. Mater. Chem. C*, 2013, **1**, 4423.
4. Y. Li, P. Sonar, S. P. Singh, M. S. Soh, M. van Meurs and J. Tan, *J. Am. Chem. Soc.*, 2011, **133**, 2198-2204.
5. J. Lee, M. Jang, S. M. Lee, D. Yoo, T. J. Shin, J. H. Oh and C. Yang, *ACS Appl. Mater., Interfaces* 2014, **6,** 20390-20399.
